# Evaluating models of consent in changing health research environments

**DOI:** 10.1007/s11019-022-10074-3

**Published:** 2022-03-14

**Authors:** Svenja Wiertz, Joachim Boldt

**Affiliations:** Department of Medical Ethics and the History of Medicine, University of Freiburg, Stefan-Meier-Str. 26, 79104 Freiburg, Germany

**Keywords:** Informed consent, Broad consent, Biobank, Big data, Ethics, Research ethics

## Abstract

While Specific Informed Consent has been the established standard for obtaining consent for medical research for many years, it does not appear suitable for large-scale biobank and health data research. Thus, alternative forms of consent have been suggested, based on a variety of ethical background assumptions. This article identifies five main ethical perspectives at stake. Even though Tiered Consent, Dynamic Consent and Meta Consent are designed to the demands of the self-determination perspective as well as the perspective of research as a public good, they are still also criticized from both perspectives. In addition, criticisms based on concerns of justice, participation and democratic deliberation, and relational concerns have been levelled at each of the models. As all of these perspectives have valid points to make, the task at hand lies in balancing these ethical perspectives. What constitutes an adequate balancing depends on contextual factors. These factors include digital infrastructure and digital literacy, data safety regulation, good scientific and clinical practice, transparent debates on ethically relevant features of research, social inequalities, anti-discrimination laws and practices, trust in health care institutions and recognition of patient preferences, and consensus on unethical research. We argue that the role of context in determining acceptable models of consent puts the ethical importance of models of consent into perspective. Since altering contextual factors can help to live up to the ethical concerns at stake in debates about models of consent, opting for such a shift of focus comes without ethical loss.

## Informed consent in changing medical research environments

Informed consent has been established as a necessary prerequisite for research involving identifiable human samples or personal health related data. Traditionally, it is understood as study specific consent that is given by research participants after being adequately informed about the aims, benefits and burdens of a particular study. The emergence of biobanking and large medical databases holds the promise of new opportunities for more and better medical research. In this context, however, obtaining consent from all patients whose samples and data are stored prior to every single study would be impractical and burdensome, sometimes even impossible. Insisting on study specific consent would thus severely constrain research opportunities. From a variety of different perspectives, models of Broad Consent, Tiered Consent, Open Consent, Dynamic Consent and Meta Consent have been suggested to appropriately address this challenge.

In the following, we argue that different consent models reflect different, specific ethical concerns to varying degrees. We start with some remarks on the relation of the European General Data Protection Regulation on issues of consent and the ethical debate on models of consent. (Sect. 2), We then present some of the suggested models of consent (Sect. 3), identify ethical concerns incorporated in these models (Sect. 4), and set out to give a structured overview of pros and cons, based on the ethical concerns identified, of each of the models (Sect. 5). This overview leads us to conclude that no single model of consent can address all of the ethical concerns at stake adequately. As a balancing of ethical perspectives becomes inevitable (Sect. 6) contextual factors need to be taken into account (Sect. 7).

## The GDPR and the ethical debate on models of consent

Governance of research on sensitive personal data is not only an issue of soft law and ethics. In the European Union, for example, the General Data Protection Regulation (GDPR) provides a legal framework that sets forth conditions for legitimate processing of sensitive data. Three of those conditions are potentially relevant for scientific research on sensitive personal data. One of them is consent, the two others are public interest and the necessity of the processing of special category data for scientific research (Hallinan 2020).

With regard to consent, it is subject to scholarly controversy whether broad consent, as traditionally understood and practiced in biobank research, is acceptable under the GDPR regimen, or whether GDPR consent must rather be understood along the lines of narrower consent models such as dynamic consent (Hallinan 2020, Gefenas et al. 2021). As a matter of principle, should this controversy come to an end either by scholarly consensus or the force of jurisdiction, the resulting allegation might still be ethically debatable. For the time being, though, in light of this ongoing controversy, determining ethically advisable consent models is important in order to ensure good practice.

This applies all the more, given that sidestepping consent and taking resort to public interest or the GDPR research exemption implies losing the ethical and social advantages of consent. Consent allows research participants to be informed about research purposes, compare them to their own values, and make those purposes their own and assist them by supplying their data if they wish. Consent thus is an important part of realizing equity (Gefenas et al. 2021).

## Models of consent

Since the second half of the 20th century, informed consent has been regarded as a hallmark of ethically justifiable medical research involving humans. Obtaining consent expresses respect for the right to self-determination of research participants. It ensures that patients can make well-informed decisions to participate - or not to participate - in the study proposed, in accordance with their values, and aware of the risks and potential benefits of the study. As this presupposes detailed information about the study, its methods, purposes, etc., informed consent needs to be obtained prior to and for each single study. This is the traditional model of S*pecific Informed Consent* (McGuire and Beskow 2010; Capron 2018; Manson and O’Neill 2008; Mikkelsen et al. 2019).

*Broad Consent*, in contrast to Specific Consent, asks research participants to consent to multiple future studies the nature and specificities of which are not known at the time of consenting. Information about aims, risks and potential benefits thus is provided in rough outline only, for example by naming general objectives of the projects, invoking guidelines all future research projects are obliged to follow, and by informing about risks that are common to all these projects. Broad Consent has widely been defended as the appropriate model of consent for biobank and health data research (Mikkelsen et al. 2019; Hansson et al. 2006). Depending on context, its actual ‘broadness’ can vary significantly and no common standard can be found in the debate for marking the difference between Broad Consent and *General Consent* – a form of consent where no restrictions on research aims and guidelines to be followed are part of the agreement.

The model of *Tiered Consent* can be regarded as a compromise between Specific and Broad Consent. The main difference between Broad Consent and Tiered Consent is that the latter provides the possibility to choose the broadness of the individual consent. In the consent procedure, several questions are asked to determine the scope of the individual consent, which can range from only study specific consent to broad consent to general consent. Tiered Consent is sometimes also referred to as Multi-Layered Consent. Typically, the options offered are formulated along the lines of issues supposed to be of individual or societal ethical relevance. For example, questions address types of disease the future research may be dealing with, options of sharing data with other institutions, and options regarding the return of information about incidental findings (Nembaware et al. 2019; Bunnik et al. 2013; Salvaterra et al. 2008; Mikkelsen et al. 2019).

*Dynamic Consent* “describe[s] personalised, online consent and communication platforms” (Budin-Ljøsne et al. 2017). It is not in itself a model of consent, but the online platform is meant to facilitate the consent process as well as ongoing communication between researchers and participants. Obtaining and giving consent may be organized much more efficiently through such a platform as compared to paper-based consent forms. It is usually suggested to implement such a platform at national (more precisely, health system) level. The option of implementation on a smaller or larger scale is not in principle excluded, but would be less efficient. Dynamic consent platforms are the basis for Dynamic Specific Consent and Meta Consent.

In a model of *Dynamic Specific Consent*, online platforms would allow research participants to view information in a format of their choice, adequate to their level of education and interest. An option to pose further questions to researchers could be provided. Information should not only be provided at the onset of a new study, but should be updated regularly to keep participants informed (Prictor et al. 2018; Budin-Ljøsne et al. 2017; Kaye et al. 2015). Participants could give their consent to individual studies from home, at any convenient point in time, after a request has been addressed to them through the platform. Sufficient understanding of the information provided could be checked through use of multiple-choice tests. Researchers would not have to deal with participants individually but could focus on preparing adequate information material and distribute the material easily to all potential participants.

The *Meta Consent* model builds on the same idea as the model of Dynamic Specific Consent. Instead of setting a fixed form of consent for all research participants, however, it suggests to set up the platform in a way that allows individuals to give either Specific or Broad Consent, depending on personal preferences. As in Tiered Consent, categories are suggested to allow for different consent settings in regard to different areas and forms of research. The individual consent preferences would be managed by the platform and could be changed at any time. As with Dynamic Specific Consent, researchers would have to provide the required information and request the system to contact potential research participants. The system would handle these requests automatically in accordance with participants’ preferences (Ploug and Holm 2015, 2020, 2019; Kaye et al. 2015).

## Ethical concerns in the debate about informed consent

When one argues ethically for or against a model of consent, one does so on the basis of ethical perspectives and concerns. We introduce the umbrella terms “perspectives” and “concerns” here in order to cover the realm of normative orientations at different levels of abstraction, from principles to values, to theories, also including rather loose concepts such as “relational concerns” that may otherwise stand in need of further analysis in order to identify the rock bottom of ethical principles at stake. A variety of concerns comes to the fore in the debates on models of consent. The following overview is intended to capture the most important concerns.^1^

### Protection of individual self-determination

One of the most central concerns in research ethics since the second half of the 20th century has been the protection of individual rights to self-determination.^2^ As the Nuremberg Code of 1947 famously states with regard to medical research on human subjects:

“All agree, however, that certain basic principles must be observed in order to satisfy moral, ethical and legal concepts: 1.The voluntary consent of the human subject is absolutely essential. This means that the person involved should have legal capacity to give consent; should be so situated as to be able to exercise free power of choice, without the intervention of any element of force, fraud, deceit, duress, overreaching, or other ulterior form of constraint or coercion; and should have sufficient knowledge and comprehension of the elements of the subject matter involved as to enable him to make an understanding and enlightened decision.” (Nuremberg Code (1947) 1996).

Traditionally, the most important individual right at stake in the context of medicine is the preservation of bodily integrity. Biobanking and data-based research pose little danger in that regard, but they do make use of personal health related data and potentially uncover new health related information. This may result in harm for research participants when this information reaches insurance companies or employers (Kasperbauer et al. 2018), or it may not be in line with individual preferences and values and thus conflict with self-determination (Mikkelsen et al. 2019), or it may simply be regarded to infringe on privacy. Biobank and data-based research thus may compromise rights to informational self-determination, which in turn may lead to harm, compromise personal values, or infringe on privacy.

There is no consensus, though, which individual rights exactly need to be protected and how and how important concerns of self-determination should be in the ethical debate. Not all individual rights to self-determination at stake are negative rights that protect against interventions. Individuals can also be taken to have positive rights, i.e. to be enabled to realise goals and interests that are related to research. It must also be asked, then, whether models of consent sufficiently further the interests of those who want to take part in research (Caulfield 2007; Hansson et al. 2006; Hummel et al. 2020; Hummel et al. 2018; Christensen 2012).

In any case, protecting individual rights to self-determination is one of the most important ethical demands a model of consent has to live up to. The central question posed by all arguments addressing this concern is: Are all relevant individual rights to self-determination respected by a given model of consent? If individual rights are compromised by consent models, can a sufficiently strong justificatory reason be given?

### Medical progress as a public good

The most obvious reason for lowering standards of informed consent is that medical progress is a public good, because it constitutes a means to improve the health of a community and future generations. On this ground, one can argue for a moral obligation to research:

“Most, if not all diseases create needs, in those who are affected, and in their relatives, friends, and carers and indeed in society. Because medical research is a necessary component of relieving that need in many circumstances, furthering medical research becomes a moral obligation.” (Harris 2005).

Out of concern for the good of society one should thus lower costs where acceptable and facilitate international research cooperation, among many other similar actions (Manson 2019a; Grady et al. 2015; Jahns et al. 2019). Regarding models of consent, one should accordingly be concerned with removing unnecessary obstacles to research. The main questions addressed to any model of consent from this perspective are thus: Is socially valuable research made possible? Is the consent process an obstacle to this kind of research?

### Participation and democratic legitimization

Concerns about democratic legitimacy and rights to participation are the foundation of another perspective in the debate, resulting in requests for deliberative processes and broader options for participation:

“The principle of participation requires decision makers not merely to imagine how people with morally relevant interests ought to expect data to be used but to take steps to discover how they do, in fact, expect data to be used and to engage with those expectations. The participation of people with interests at stake in the design of data initiatives gives decisions a strong claim to legitimacy. Independently of the outcome, participants, and the wider public, are more likely to accept the process as being a fair and respectful way of resolving any differences between them with regard to decisions that may affect them all.” (The Nuffield Council on Bioethics 2015).

What constitutes the public good cannot be determined independently of the interests and preferences of citizens. This is the ethically important rationale behind including citizens in debating and defining what kind of medical research should be prioritized, for example. Likewise, participation in processes of choosing, implementing and refining a consent model add to the ethical acceptability of any such model. From this perspective, the central question is not which form of consent is in and of itself best. Rather, the focus shifts to asking who is and who should be involved in decisions about implementing models of consent. This perspective underlines that it is not features of consent or research alone, but also practices of regulating consent and research that determine its legitimacy. Even though this is mainly a general concern about social contexts of research, models of consent have themselves occasionally been rejected or defended by reference to opportunities of participation (Arnason 2004; Gould 2019).

### Considerations of justice

Concerns about justice affect the discussion of appropriate models of consent insofar as opinion studies have shown that a willingness to share data and consent broadly to research is not distributed equally among social groups. Differences between black and white people, men and women, the rich and the poor have been revealed (Brown et al. 2016; Moodley et al. 2014; Prictor et al. 2018; Garrison et al. 2016).

This can be understood as leading to an unjust situation directly, for example if some social groups have the resources and standing to critically select studies they wish to participate in while others who lack relevant capacities are left to accept all studies offered, even dubious ones. In cases or contexts where data safety regulation is lax, this is a relevant concern.

In addition, an indirect consequence of unequal participation, often cited in the literature, is an increase of existing inequalities in health care:

“If biobanks are not appropriately reflective of the wider population, nor will the research findings derived from them be representative. This raises the risk that research products reaching the clinic will only be relevant for certain sections of the population, which could further exacerbate inequity and health inequalities.” (Prictor et al. 2018).

This is an important ethical issue in any society with significant existing health inequalities as well as on a global level – where such inequalities certainly do exist (Arcaya et al. 2015; Cash-Gibson et al. 2021; Shannon et al. 2019).

The main questions that considerations of justice address are, accordingly: do informed consent procedures lead to unjust distribution of the benefits and burdens of research between different social groups?

### Relational considerations

Last but not least, relations between research participants and researchers (or research institutions) have been addressed in the debate (McGuire and Beskow 2010; Bromley et al. 2020). Trust and respect are the relational values most commonly named (Lentz et al. 2016; Resnik 2018; Master et al. 2015; Campbell 2007). Sometimes, these properties are regarded as goods in themselves, sometimes they are considered as a means to upholding societal acceptance of research and individuals’ willingness to participate:

“Lastly, obtaining consent makes transparent decisions about donating and researching biospecimens. Such transparency can promote public trust, and the ongoing viability of research with stored samples. These considerations suggest a strong ethical rationale for obtaining donor consent for the future research use of biospecimens.” (Hansson 2009).

Thus, although trust and related relational properties are not a valuable potential outcome of research (in the way improvements in health care are), they are necessary prerequisites of research. The consequences of a loss of trust for the willingness to participate in research have to be taken seriously as they directly affect the potential for future research. Furthermore, in the long run lack of trust in research may even lead to a lack of trust in medical institutions at large, thus undermining the quality of public health care.

The relational perspective thus rightly asks how consent models shape the relationship between researchers and participants. It considers how different models of consent could support or undermine trust in medical research and health care institutions.

## Evaluating models of consent

### Specific consent

From the perspective of individual self-determination, Specific Informed Consent is defended as the established standard that does justice to the human right of self-determination and shows appropriate respect for individual autonomy, since it ensures that research participants know what they are asked to consent to, and since it enables them to take an informed stance on whether to consent or not (Caulfield and Kaye 2009). Prima facie, this is a strong argument in favour of Specific Informed Consent.

This judgement has been called into question by pointing out a shift in relevant risks in biobanking and data based research as compared to traditional clinical research. Mikkelsen et al. (2019) argue that informational risk, the most relevant risk in this context, depends on data safety regulations of the data registry and operation guidelines of the biobank much more than on the particular research carried out. Hence, one would neither *need* to be informed about study specificities, nor would study specific consent be *sufficient* to inform about these risks.

While this appears correct as it stands, this argument implicitly reduces the value of self-determination to being able to avoid harm. It does not take into account that self-determination also includes being able to decide which research purposes one may want to support, for example.

In addition, the claim that informational risks are the only relevant risks of biobank and data registry research appears false. Ploug, for example, invoking relational concerns, points out that anxiety and insecurity about the use of data may reduce trust in health care professionals, leading to patients withholding information, and thus decreasing quality of treatment (Ploug 2020).

Most importantly, Specific Informed Consent is criticised for hindering medical progress, as biobanks and data registries would have to invest significant resources to contact potential study participants, given the large number of participants involved. It is thus rejected as too onerous, expensive and time consuming (Grady et al. 2015; Hansson et al. 2006; Mikkelsen et al. 2019; Budin-Ljøsne et al. 2017). This is a relevant and important concern. It is not a refutation of the value of self-determination, though. What is called for, in effect, is an appropriate compromise between the demands of the two perspectives.

Some authors have also pointed to the danger of ‘consent fatigue’ on the side of research participants: If persons are asked to review similar kinds of information again and again they are likely to stop paying attention and to give consent in the form of a merely habitual act. If this happens due to too many requests for Specific Informed Consent, the consent procedure fails to protect self-determination (Mikkelsen et al. 2019; Cambon-Thomsen 2004; Ploug and Holm 2013). Again, this does not compromise the value of self-determination as such. Rather, it calls into question whether Specific Informed Consent can effectively realize self-determination. It is a call for alternative ways to safeguard self-determination, not a rejection of concerns based on self-determination.

Taking these arguments into account, Specific Informed Consent does not appear as the best possible model to satisfy the perspective of medical progress, nor does it necessarily appear as the right answer to a perspective of self-determination in modern research contexts.

### Broad consent

Broad Consent has been criticized for not protecting self-determination adequately in different ways. Some authors argue that it is simply not sufficient to protect autonomous decision making, as the information provided is not adequate for such decisions. It is possibly insufficient in informing about potential future risks (Hofmann 2009), or it might be insufficient since it does not provide *control* over one’s own data, while truly respecting self-determination would imply providing such control (Caulfield 2007).

If taken at face value, arguments of this kind amount to plain rejections of Broad Consent. In comparison to other models of consent, Broad Consent certainly provides least information and least control over one’s data and specimen. Nonetheless, and at closer sight, if one, for example, supplies Broad Consent with publicly available regular information on ongoing research and an option to withdraw consent, Broad Consent may be able to accommodate these concerns of control and information. Changing contextual factors thus could render Broad Consent acceptable from a self-determination perspective.

In any case, respect for self-determination is also referenced in the debate in order to argue *in favour* of Broad Consent. Given that no individual must participate in research, legally restricting models of consent that appear acceptable to some is criticised as paternalistic and an undue restriction of the individual right to participate in research (Campbell 2007; Hofmann 2009). Arguments of self-determination do thus not fall on only one side of the debate. Although consent models such as Dynamic or Meta Consent are not necessarily more cumbersome to handle than Broad Consent for individuals willing to participate in research, this argument shows that Broad Consent cannot be simply dismissed on grounds of self-determination concerns.

Another concern reiterated in the debate addresses the validity of Broad Consent over time. Including the option to withdraw consent is usually supposed to ensure that Broad Consent stays valid over time. If, however, it is plausible to assume that at least some research participants may simply have forgotten about their participation over the course of years, the absence of a withdrawal alone might not ensure the validity of consent (Hofmann 2009; Mikkelsen et al. 2019; Ploug and Holm 2020).

Furthermore, consent might be invalidated by differences in interpretation – if research characteristics are outlined too broadly and research contexts, in accordance to which these characteristics need to be interpreted, change (Ploug and Holm 2020). This poses a practical challenge for researchers and database operators when making decisions on whether a specific set of data can be used for a specific study. It also poses a problem under the relational perspective: If participants become aware through public media that samples and data were used for research contradicting their values, public trust may easily be lost (Caulfield 2007). Again, introducing contextual changes such as accompanying Broad Consent with regularly updated, publicly available information on ongoing research may mitigate these concerns.

Broad Consent is often seen as the model that is best suited to further medical progress and is thus best suited to further the public good and public interest. The perspective of medical progress arguably supplies the strongest argument in favour of Broad Consent.

Nonetheless, as was remarked above, defining the public good entails taking into account the preferences and interests of citizens. Caulfield and Kaye (2009) convincingly argue from a democratic perspective that the question of whether research is in the public interest cannot be decided either by researchers themselves or by ethical review boards – the public alone should be considered competent to decide what is and what is not in the public interest. This call for democratic participation does not count against Broad Consent as such, but it does imply incorporating options of participation in choosing, implementing, and revising Broad Consent.

From a perspective of distributive justice, concerns have been raised that Broad Consent, while acceptable to some, is not equally acceptable to all. Since acceptability varies across different groups, already disadvantaged minorities might be further disadvantaged by being underrepresented in medical research (Garrison et al. 2016; Prictor et al. 2018). This is certainly a valid concern. Nonetheless, how pressing it is depends on social context: The more socially inhomogeneous a society is, and the more stigmatized certain groups are, the more of an issue exclusion from research will become. What is more, these concerns apply not only to Broad Consent, but to all other consent models as well. Online consent platforms may be a barrier for research participation, and so may laborious Specific Consent forms. This implies, firstly, that which consent model is best suited to accommodate the perspective of justice depends on the specific social characteristics of the society and groups involved. Secondly, justice concerns regarding participation in research can always also be addressed, and perhaps be better addressed, by other means than the choice of a consent model.

### Tiered consent

Tiered Consent is generally defended as a model that addresses interests of individual self-determination better than Broad Consent, as in this model the consent given can be either broad or more restricted, depending on the choice of the research participant. Use of this model has been recommended for research in third world countries, especially in Africa, where a Broad Consent approach might seem particularly problematic in light of a history of colonialism and exploitation (Tiffin 2018; Nembaware et al. 2019).

Limited elements of the idea of tiered consent have also found their way into practice when study participants are, for example, asked to specify whether they consent to the use of their samples and data only in research conducted by public research institutions, or whether private companies may use the material as well. Following the GDPR, research participants in EU countries can also prohibit or allow use of their data in countries with differing legal data safety provisions.

One particular problem with Tiered Consent is how to store the consent given for a particular sample and set of data. If no clear process is implemented, it cannot be guaranteed that the individual preferences will actually be adhered to when future research studies are carried out. It has been suggested that standardized ontologies for labelling data samples would be a necessary requirement for a large-scale implementation of Tiered Consent (Nembaware et al. 2019). Digital solutions for storing consent information certainly seem the right way forward for enabling reuse of data as well as allowing for international research cooperation where this option is covered by the consent given.

Objections have been raised against the idea of categorizing research in the way it would be necessary for Tiered Consent. The model has been declared as necessarily unjust because any system of categorization could not but reflect a specific set of values (Mikkelsen et al. 2019).

One may doubt that this actually is the case since it is always possible to tick the “Specific Consent” option, which allows one to streamline one’s choice project per project.^3^ This option would make it possible to adhere to any personal set of values even if it is not mirrored in the tiered choices that are explicitly named in the Tiered Consent form. This option comes at a price, though: It re-introduces the laborious procedure of Specific Consent for both researchers and participants.

Besides the Specific Consent option within Tiered Consent, another way to ameliorate the problem of pre-defined categories would be by incorporating participation. If the set of options one settles upon is justified via public participation and decision-making, it may still not accommodate every individual’s preferences, but it may nonetheless cover the preferences of a large majority of participants.

From a perspective primarily concerned with medical progress, Tiered Consent requirements have been rejected as cumbersome and time consuming, just as Specific Consent, especially where complex consent documents would have to be translated into several languages (Tiffin 2018; Mikkelsen et al. 2019). This argument certainly holds true in comparison to Broad Consent. At the same time, compared to Specific Consent, the amount of time and work required would be reduced. In any case, implementing Tiered Consent on a digital platform, as proposed in the model of Meta Consent, would further ameliorate the burden of time and work without compromising the higher level of self-determination offered by Tiered Consent compared to Broad Consent.

### Dynamic specific consent

Dynamic Specific Consent has been presented as a means to make Specific Informed Consent possible in the context of biobanking by using an online platform for the consent process. Following the idea of Specific Informed Consent it has been defended as a way to respect research participants’ autonomy by allowing them to decide on a case by case basis in which studies they wish to take part (Budin-Ljøsne et al. 2017; Kaye et al. 2015; Dankar et al. 2020).

This advantage has been called into question by pointing out the danger of ‘consent fatigue’: If too many requests are addressed to a person in a short amount of time, it becomes likely that participants will fall into a habit of routinely clicking to agree without adequately taking note of the information provided. In this case consent might no longer be sufficiently informed (Ploug and Holm 2015). As the clicking habits concerning buttons for acceptance of data privacy terms and conditions for webpages or mobile apps indicate, this may very well be a reasonable concern.

Considering cost and effort for researchers, Dynamic Specific Consent seems better suited to the biobank and data registry context than standard Specific Informed Consent, since it facilitates the consent process in a number of ways: As information is made available online, researchers have to invest less time in sharing information with participants. No paper records need to be kept. Recruitment for future studies as well as recontact for additional data is handled by the platform (Kaye et al. 2015; Budin-Ljøsne et al. 2017).

While even defenders of the model have admitted that the cost for implementation is likely to be significant, it is expected that once the platform has been implemented, costs for its upkeep should be comparatively low, especially if the tool is made available on a national level, as commonly suggested (Budin-Ljøsne et al. 2017). From a perspective of justice, it has on the one hand been pointed out that the model can make information more accessible to many by providing it in different formats and languages. On the other hand, accessibility also is one of the central justice related concerns: It is dependent on digital literacy and availability of digital equipment. Here, a danger of aggravating differences often referred to under the term of a ‘digital divide’ has been identified (Budin-Ljøsne et al. 2017; Prictor et al. 2018).

From a perspective of participation, often mixed with references to a relational perspective, it has been stressed that Dynamic Specific Consent offers opportunities for ongoing engagement: Dynamic Specific Consent is supposed to allow for an active engagement of participants and ongoing communication in both directions. It is claimed that it can thus reduce mistrust and improve research literacy among participants (Budin-Ljøsne et al. 2017; Kaye et al. 2015; Dankar et al. 2020; Prictor et al. 2018). Whether this promise would actually be fulfilled seems to depend largely on the specifics of implementation of such a platform.

Dynamic Specific Consent thus offers a high level of self-determination while being less time-consuming to handle than Specific Informed Consent. It may also incite engagement with research. Accessibility with regard to language and formats of the information can be high, although the digital implementation imposes new barriers to those not acquainted with digital devices. As a drawback, Dynamic Specific Consent may lead to superficial consent when participants receive many requests to participate in research in a short time.

### Meta consent

The Meta Consent model, like Dynamic Specific Consent, is defended as an alternative to Broad Consent as it allows for a more adequate respect of individual autonomy. It is argued that in this model individual rights and interests are respected as comprehensively as in the model of Dynamic Specific Consent, since research subjects can choose the specific consent option. As the model does allow for broader consent options as well, it is at the same time better suited to prevent consent fatigue (Ploug and Holm 2015, 2019). Broad Consent becomes acceptable in this framework as an option chosen by the participants themselves, ongoing information ensures awareness of the scope of research, and participants can change preference settings at any time (Ploug and Holm 2019, 2020).

The model shares many of the advantages of Dynamic Specific Consent as a digital platform at health system level. In addition, it has the further advantage to allow participants to choose Broad Consent for wide areas of research that they generally wish to support.

As the Meta Consent model relies on the idea of offering categories in a similar way to the model of Tiered Consent it also faces the challenge of categorization, with the advantage that this problem could be addressed at platform level and ideally in a process that allows for direct patient participation in determining such categories.

At the same time, Meta Consent also faces some of the challenges Dynamic Specific Consent has to deal with: The costs for implementation of Meta Consent would certainly be higher compared to Broad Consent. How significant they would actually be is a contested issue (Ploug and Holm 2019; Manson 2019a, 2019b).

In addition, and most importantly, the challenge of accessibility and the risk of furthering a digital divide applies to Meta Consent as well as to Dynamic Specific Consent. Depending on societal context, this challenge may be more or less pressing. In societies with a significant number of citizens who would be excluded from participating in research, supplementary means to obtain consent could be implemented, such as assisted completion of the digital form at hospitals or physicians’ offices.

## Balancing perspectives

As the overview of ethical evaluations of informed consent models shows, and this is not much of a surprise, protecting individual rights to self-determination, on the one hand, while allowing for the realization of the public good of medical progress, on the other hand, is the conflict at the centre of the debate. From the perspective of self-determination, Specific Informed Consent is the consent model of choice. Given that biobank and data based research does not infringe on bodily integrity, but on privacy and informational self-determination, the call for Specific Consent may be attenuated. Still, all else being equal, any alternative to Specific Consent diminishes the ability to decide which kind of research one wants to support with one’s personal data and thus diminishes the ability to self-determination. Furthering medical progress, by contrast, leads on to Broad Consent or even General Consent as the favourable model of consent. The laborious task of having to obtain specific consent appears, from this perspective, as an inappropriate obstacle to research.

Taking this schematic conflict as a point of reference, Tiered Consent, Dynamic Consent, and Meta Consent all can be understood as attempts to mediate between the two concerns. As the overview of arguments for and against the models also shows, each of these models still is, in fact, criticized regarding both concerns. Dynamic and Meta Consent platforms dispense with the need to laboriously obtain individual specific consent, but these platforms still have to be established and maintained and thus require funding that could otherwise, in principle, promote research. At the same time, these platform solutions involve pre-established categories in relation to which users chose their preferences. These categories may not reflect the actual user preferences, though. Not making use of the offered categories at all by choosing study-specific consent is an option for participants, but one that comes at the cost of time-consuming checking of each single project.

The overview of ethical concerns and arguments for and against consent models also shows that reconstructing the debate as a tug war between two perspectives is an oversimplification. Besides self-determination and research as a good, other concerns play a role as well in the debate on consent models. Justice, participation and democratic decision-making, and relational concerns highlight a variety of drawbacks and merits of each consent model.

Given the tension of the perspectives of self-determination and progress, and given the variety of additional ethical concerns directed at consent models, no single consent model can lay claim to be ethically best under all circumstances. Choosing a model becomes a matter of finding an acceptable compromise, i.e. of balancing ethical perspectives.

What constitutes an adequate balancing of perspectives depends, inter alia, on institutional, political, and social context. For example, if institutional data safety practices live up to the highest standards and research participants are socially homogenous and can all be assumed to back up medical research, broad consent may be the model of choice. Similarly, if a consent platform can be set up as an extension of a pre-existing health care platform with assured accessibility and at low cost, this would count in favour of Meta Consent or Dynamic Consent Models. If a participatory process for determining normatively relevant categories of research can be established, Meta Consent would offer clear advantages over Dynamic Specific Consent.

As these advantages are dependent on a number contextual factors, the resulting landscape of models of consent may be varied, differing from country to country, or, to be more precise, from one socially and legally homogenous region to another region. This, in turn, constitutes a challenge to large-scale international research.^4^ One solution to this problem would be to define and establish digital interfaces that allow international research projects to request and be fed with data in accordance with local consent regulation. National or local data use and access committees could act as gatekeepers who supply the interface and release data. Another solution would be to align contextual factors. In fact, the GDPR may be seen as such a tool to align at least the legal part of data safety, as mentioned below, at an international level.

## From models of consent to contexts of consent

The ethical concerns that are at stake in evaluating models of consent are not only relevant for consent but for a wide field of political, governance and institutional contexts of consenting procedures. These contexts can render a consent model ethically acceptable that would in other contexts be deemed inacceptable. Based on the analysis of the debate as provided above, relevant contextual factors include:

(1) Digital infrastructure and digital literacy. If consent platforms can take advantage of pre-existing digital infrastructure, development and maintenance costs are lower. If digital literacy among potential research participants is high, consent platforms do not lead to exclusion of (groups of) participants.

(2) Data safety regulation: If a high level of data safety is established, and data access and usage comply with data protection rules, lower levels of the specificity of informed consent become acceptable. For example, the GDPR and GDPR data protection rules constitute, if adhered to, a comparatively high level of data safety, which, in turn, justifies lower levels of the specificity of consent.

(3) Established standards and safeguards of good scientific and clinical practice: If awareness of norms of good scientific and clinical practice is high, trustworthiness of research institutions is increased and the details of the consent procedures are of lesser importance.

(4) Transparent debates on ethically relevant categories of research: If public debates on ethically problematic aspects of research exist, relevant features of research can easily be identified, and thus Tiered Consent and consent platforms can mirror these categories and go hand in hand with many individual values.

(5) Social inequalities and safeguards against them: The less social inequalities exist, the lower is the risk of unequal distribution of health services and research benefit and burden. As a consequence, the need to identify consent models that appeal to vulnerable groups and to establish specific consent becomes less urgent.

(6) Anti-discrimination laws and practices: If anti-discrimination laws and practices are in place and effective, the need for consent processes that are designed to protect from discrimination become less urgent.

(7) Trust in health care institutions: If trust in the healthcare system is high, as a result of good data protection and recognition of patient values and interests, the specificity of informed consent can be lowered.

(8) Consensus on unethical research: If societal consensus exists on what is to be regarded as unethical research, and if this kind of research is forbidden and the ban is observed by researchers, trust in research will be fostered.

All these contextual factors can be taken as they are, and be set to use in determining acceptable models of consent. Generally speaking, the higher the standard of data protection is, the more institutional and political safeguards are in place and accountability is guaranteed, the more equal a society is and the higher public trust in health care, the less specific consent procedures can become, especially if introduced through democratic means. Conversely, if social inequalities are high, if social trust in health research is limited, and if questionable research practices are permitted, stressing specific consent becomes more important. Furthermore, the ethical acceptability of digital solutions depends primarily on levels of availability and familiarity with digital tools. They can provide advantages when they are accessible to all social groups.

In addition, the role of context in determining acceptable models of consent also implies that instead of focusing on models of consent, one may also focus on improving these contextual factors. Altering contextual factors can help to live up to the ethical concerns that have come to the fore in the debate. Opting for such a shift of focus may help to put the ethical importance of models of consent into perspective.

## Conclusion

None of the consent models satisfies fully both the demands of the individual rights perspective and of the perspective of research as a public good. Even though Tiered Consent, Dynamic Consent and Meta Consent are designed to fit both perspectives, they are still met with criticism from both sides. In addition, valid criticisms based on concerns of justice, participation and democratic deliberation, and relational concerns have been levelled at each of the models.

In the light of these criticisms, and given the fact that all these perspectives appear ethically relevant, it becomes impossible to declare one model ethically best under all circumstances. Given the tension of the perspectives of self-determination and medical progress, and given the variety of additional ethical concerns directed at consent models, no consent model can lay claim to be ethically uncontested. Instead, the task at hand is to identify an acceptable compromise, which is to say to balance ethical perspectives.

What constitutes an adequate balancing of perspectives depends, inter alia, on institutional, political, and social context. Relevant contextual factors include digital infrastructure and digital literacy, data safety regulation, good scientific and clinical practice, transparent debates on ethically relevant features of research, social inequalities, anti-discrimination laws and practices, trust in health care institutions and recognition of patient preferences, and consensus on unethical research.

Generally speaking, the higher the standard of data protection is, the more institutional and political safeguards and are in place and accountability is guaranteed, the more equal a society is and the higher public trust in health care, the less specific consent procedures can become, especially if introduced through democratic means.

Taking these factors into account in different societies may lead to different ethical recommendations regarding models of consent. At the same time, the role of context in determining acceptable models of consent puts the ethical importance of models of consent into perspective. Since altering contextual factors can help to live up to the ethical concerns that have come to the fore in the debate on informed consent, too, opting for such a shift of focus comes without ethical loss.

## Data Availability

not applicable.

## Footnotes

1 We take all of the introduced perspectives to provide insight into relevant and valid ethical concerns. In the following, we point out what we deem to be plausible and important claims made on the basis of these perspectives. An in depth defence of each perspective, however, is beyond the scope of this paper.

2 The focus on individual rights to self-determination might seem to be tied to a perspective of deontological ethics. But this is not necessarily the case. The Nuffield Council report, for example, points to a legal right to privacy deeply rooted in utilitarian and liberal tradition in “defence of a sphere of self regulation and private action against interference by others, and especially by public authorities” The Nuffield Council on Bioethics (2015).

3 We thank an anonymous reviewer for pointing this out to us.

4 We would like to thank an anonymous referee for pointing this implication out.
